# Conversation Therapy with People with Aphasia and Conversation Partners using Video Feedback: A Group and Case Series Investigation of Changes in Interaction

**DOI:** 10.3389/fnhum.2016.00562

**Published:** 2016-11-07

**Authors:** Wendy Best, Jane Maxim, Claudia Heilemann, Firle Beckley, Fiona Johnson, Susan I. Edwards, David Howard, Suzanne Beeke

**Affiliations:** ^1^Department of Language and Cognition, Division of Psychology and Language Sciences, University College LondonLondon, UK; ^2^Homerton University Hospital NHS Foundation TrustLondon, UK; ^3^Psychology and Clinical Language Sciences, University of ReadingReading, UK; ^4^Speech and Language Sciences, School of Education, Communication and Language Sciences, University of Newcastle upon TyneNewcastle upon Tyne, UK

**Keywords:** aphasia, conversation, intervention, video feedback, agrammatic aphasia, therapy, outcome measure

## Abstract

Conversation therapies employing video for feedback and to facilitate outcome measurement are increasingly used with people with post-stroke aphasia and their conversation partners; however the evidence base for change in everyday interaction remains limited. We investigated the effect of Better Conversations with Aphasia (BCA), an intervention that is freely available online at https://extend.ucl.ac.uk/. Eight people with chronic agrammatic aphasia, and their regular conversation partners participated in the tailored 8 week program involving significant video feedback. We explored changes in: (i) conversation facilitators (such as multi-modal turns by people with aphasia); and (ii) conversation barriers (such as use of test questions by conversation partners). The outcome of intervention was evaluated directly by measuring change in video-recorded everyday conversations. The study employed a pre-post design with multiple 5 minute samples of conversation before and after intervention, scored by trained raters blind to the point of data collection. Group level analysis showed no significant increase in conversation facilitators. There was, however, a significant reduction in the number of conversation barriers. The case series data revealed variability in conversation behaviors across occasions for the same dyad and between different dyads. Specifically, post-intervention there was a significant increase in facilitator behaviors for two dyads, a decrease for one and no significant change for five dyads. There was a significant decrease in barrier behaviors for five dyads and no significant change for three dyads. The reduction in barrier behaviors was considerable; on average change from over eight to fewer than three barrier behaviors in 5 minutes of conversation. The pre-post design has the limitation of no comparison group. However, change occurs in targeted conversational behaviors and in people with chronic aphasia and their partners. The findings suggest change can occur after eight therapy sessions and have implications for clinical practice. A reduction in barrier behaviors may be easier to obtain, although the controlled case series results demonstrate a significant increase in conversation facilitators is also possible. The rehabilitation tool is available online and video technology was central to delivering intervention and evaluating change.

## Introduction

Interventions with people with aphasia (PWA) harness technology in increasingly diverse ways to deliver individualized impairment based programs (see for example Nouwens et al., 2013; Des Roches et al., 2015; Palmer et al., 2015), rehabilitate functional communication (Bilda, 2011; Marshall et al., 2013), promote social participation (Wilson et al., 2015), provide education (Rose et al., 2010) and enable remote delivery and continued rehabilitation in the context of limited resource (van de Sandt-Koenderman, 2011; Woolf et al., 2016).

In recent years, there has been increasing interest in conversation-focused aphasia interventions underpinned by a number of different theoretical perspectives (see Simmons-Mackie et al., 2014) and with a primary focus on training the communication partner (CP). A systematic review by Simmons-Mackie et al. (2016) concludes that CP training is effective, and is likely to improve participation in conversations for people with chronic aphasia. Although perhaps not emphasizing the use of technology, from the outset conversation-focused aphasia interventions have deployed video feedback for raising CP awareness of maladaptive conversation behaviors, and for outcome measurement (see reviews by Turner and Whitworth, 2006; Simmons-Mackie et al., 2014, 2016). Approaches based on conversation analysis (CA) view video feedback as key to the success of this therapy, and make use of it to enhance the learning of PWA as well as CPs (for a review see Wilkinson, 2014). However, whilst there is evidence that video feedback results in increased self-awareness when compared to verbal feedback alone in traumatic brain injury rehabilitation targeting occupational participation in daily activities (Schmidt et al., 2011, 2012), there is limited evidence for the effectiveness of video feedback in aphasia rehabilitation. In addition, a recent survey of UK speech-language pathologists (SLPs) suggests that, even when recommended as part of a published therapy program, video feedback on conversation may not be routinely employed in clinical practice (Beckley et al., 2016).

This article reports on findings from a UK Stroke Association-funded project to evaluate a conversation-focused intervention designed to train a CP and a PWA to use self-selected strategies to facilitate conversation, and to decrease behaviors that act as barriers to conversation. Video feedback is a key feature of the intervention, Better Conversations with Aphasia (BCA; Beeke et al., 2013), which has its theoretical roots in CA (for an overview of CA-informed aphasia therapy see Wilkinson, 2014). The impact of the intervention on interaction is evaluated using videos of everyday conversation.

Participants in this study have agrammatic aphasia, classically characterized as non-fluent with associated “telegraphic” language output and word order and morphological errors, alongside relatively spared comprehension (Caramazza and Zurif, 1976; Bastiaanse and Edwards, 2004). Interventions for agrammatism, once only targeted at surface grammar, now demonstrate the benefits of rigorous theoretically motivated therapy aimed at underlying syntax (see for example, Thompson and Shapiro, 2007). Whilst there is evidence that grammatical ability in the clinical setting can be improved in this way, it has proved hard to detect carryover to everyday conversation; indeed there are few studies where this is explicitly evaluated. It may be that generalization occurs but much research has failed to capture it (Carragher et al., 2015). In the field of anomia therapy, there is evidence that work on retrieving single words can influence connected speech tasks and conversation (Conroy et al., 2009; Best et al., 2011). However, given what we understand about conversation in aphasia (for a review see Beeke, 2012), it is possible that carryover from grammatical therapies is not supported because interventions are based on utterances stripped of natural interactional context. CA research has demonstrated that utterances produced by agrammatic speakers in everyday conversation with a family member differ significantly from utterances elicited by decontextualized assessment and therapy tasks (Beeke et al., 2003, 2007; Heeschen and Schegloff, 2003).

The BCA intervention targets conversation, attempting to change behaviors known to influence the flow of conversations when a speaker has aphasia (Wilkinson, 2014). Specifically, it aims to reduce conversation barriers, such as “test” questions, asked despite the CP already knowing the answer, and to increase conversation facilitators, such as the use of gesture or writing within the turn of a PWA. In their review of conversation focused interventions, Wilkinson and Wielaert (2012) concluded there was evidence that interventions involving feedback informed by CA resulted in change in the conversational behaviors of the PWA and/or their CP, both in terms of a reduction in barriers for CPs and an increase in facilitators for both PWAs and CPs. The authors point out that for some studies the evidence was qualitative, while others combined quantitative and qualitative analyses of change; no group studies were included. Therefore this study now reports findings at group and case series level; see Thompson (2006) and Howard et al. (2015) for discussion of case and case series methodology.

When targeting conversation directly, an interesting and under researched question arises as to whether one would predict changes on formal language testing as a result. A study that speaks to this is Wilkinson et al. (2010). In this single case study there was no reported change on linguistic and cognitive tasks after conversation focused intervention. However, the participant with chronic aphasia reportedly produced more turns containing “sentences” after therapy (no further detail is supplied). More recently, Wilkinson et al. (2011) trained a person with chronic aphasia to use topic alerters such as “by the way” as a method of establishing topic. The authors reported significant improvement in the PWA’s ability to name and read aloud real words after therapy, although they do not account for this aspect of their findings. Thus there does appear to be some evidence for change on tasks tapping language impairment as a result of intervention targeting conversation, but it is very limited. One might hypothesize a mechanism whereby language tasks linked to conversation strategy use might improve, for example written picture naming in a PWA who chooses to practise the strategy of writing during conversational turns. However, with the focus of intervention squarely on interaction and not language processing, changes in reading aloud as found by Wilkinson et al. (2011) are harder to predict or explain. We selected digit span as a sensitive control task. Historically, aphasia has been viewed as an impairment of memory and it is well established that there is a correlation between performance on verbal short term memory tasks such as digit span and performance on language tasks in PWA (Salis et al., 2015). While assessment of short term memory is not without issues, digit span is widely used in research and practice, with some tests of this showing strong construct validity and predictive and discriminant validity (Murray et al., 2016). A further consideration in task selection was that the participants would be likely to be neither consistently at floor or ceiling on the task, allowing scope for change. Finally, there is a small but growing literature on short term memory intervention with PWA; several studies demonstrate improved digit span post-intervention (for an overview, see Salis et al., 2015). The knowledge that performance on this task can change with targeted treatment and also that there is no evidence, to our knowledge, of conversation intervention altering auditory-verbal short term memory, makes it a suitable control task for intervention focusing on conversation.

There is increasing momentum in applying conversation-based approaches in aphasia rehabilitation. However, the issue of measuring conversational outcomes is far from straightforward and efforts to produce quantitative outcome measures have had mixed success. Ramsberger and Rende (2002) and Ramsberger and Menn (2003) devised a reliable and valid method of analysis, but chose to focus on structured dialog rather than everyday conversation, and on information transfer without consideration of the interactional features of conversation. Kagan et al. (2004) report high inter-rater reliability (IRR) for the Measure of Skill in Supported Conversation (MSC), used to rate a CP, and the Measure of Participation in Conversation (MCP), used to rate a PWA. However, these tools are not designed to measure outcomes for therapies that target the adoption of dyad-specific trained strategies, as is the case for those underpinned by CA. Wilkinson et al. (2010) used a combination of global rating and frequency counts of individual conversational behaviors, asking a group of 15 SLPs naive to the intervention to rate conversation samples; IRR was not examined. Herbert et al. (2013) produced the “Profile of word retrieval in speech” (POWERS), also based on CA principles, which provides a reliable measure of word-retrieval in conversation (Herbert et al., 2008 explore intra- and inter-rater reliability) but the profile does not quantify strategy use. Wielaert (2016) devised a measure based on SLP judgement of paired video samples using broad CA concepts of turn-taking, repair, and conversation balance, but reported poor IRR. This literature on outcomes combined with the considerable variability found in conversation across occasions (Perkins et al., 1999) lead us to a design with multiple (six) conversations analyzed before and after intervention. Furthermore, a new conversation measure was developed using insights from POWERS and aiming to target aspects of conversation beyond word-finding. IRR for the new measure was evaluated as part of the current study.

The BCA intervention program was adapted from SPPARC (Lock et al., 2001), and aims to raise insight into the effects of aphasia on conversation, and teach strategies to allow: (i) a PWA to produce more complete, and thus successful, turns at talk, thereby increasing the likelihood of mutual understanding; and (ii) a CP to modify their responses to PWA turns, and thus enhance their partner’s chance of communicating more effectively. After video-based reflection on barriers and facilitators to their conversations, both the PWA and CP (henceforth a dyad) chose strategies to practice in coached conversations with the SLP, and in home tasks between sessions. Published findings to date are detailed single (and double) cases reporting qualitative findings alongside quantitative changes in conversation behavior (Beeke et al., 2014, 2015), and qualitative investigations of conversation change (Beeke et al., 2011), and of the therapeutic learning process, including reflections on video feedback (Beckley et al., 2013). Heilemann et al. (2014) report an investigation of treatment fidelity (TF). The findings from these studies have resulted in a free e-learning resource, also called BCA, for SLPs and for PWA and their families, which aims to increase understanding of conversations with aphasia, and promote access to conversation therapy (Beeke et al., 2013). The team has also explored the mechanisms of behavior change and “active ingredients” of BCA, using theory and tools from behavior change research in health psychology (Johnson, 2015; Johnson et al., in press). This article is the first time the data from all dyads have been compiled and analyzed at the level of the group and for the case series. While this approach does not allow for the detailed consideration afforded by single cases, which include mixed qualitative and quantitative data, it is important in providing robust group-level outcomes for conversation therapy, as called for by Wilkinson and Wielaert (2012) and Simmons-Mackie et al. (2014). We do not explore outcome in relation to background assessments or patterns in conversation. Although intervention targets differed for different dyads, in this article, we step back and examine conversational change for barriers and facilitators as a whole.

The goal of the current study is to determine the effectiveness of BCA intervention for people with agrammatic aphasia and their regular CPs at a group and case series level. Additionally the study examines the conversation strategies that change (barriers and facilitators) and draws out implications for practice and for joint goal setting.

The following questions are asked in the study: (1) Does performance on language tasks and digit span change after therapy? From the literature it was hypothesized that language tasks might show change but that digit span would remain stable; (2) Does the number of conversation facilitators before intervention differ from that after intervention?; and (3) Does the number of conversation barriers before intervention differ from that post intervention? The study also explores intervention fidelity and IRR for the video-based measure of conversation.

## Materials and Methods

### Participants

The study was granted multi-site UK National Health Service (NHS) ethical approval from Cambridgeshire 1 Research Ethics Committee (project ID 08/H0304/40) and written informed consent was obtained from all participants. Participants were people with agrammatic aphasia who had: (i) a left CVA at least 6 months prior to involvement in the study; (ii) no significant hearing loss, cognitive or psychiatric disorder which would affect participation; (iii) a spouse, family member or friend who communicated with them daily and was keen to participate; and who were (iv) not having speech and language intervention at the time of the study. Referrals came from the geographical areas of Greater London, the South, and South East of England, and were made by NHS SLPs, and by the coordinators of university aphasia clinics and stroke support groups run by charities. A diagnosis of agrammatism was made by the project team from an audio recorded picture description task, and free conversation with the research SLP.

### Intervention

#### Description of Intervention

Weekly therapy sessions of approximately 1.5 h each took place at participants’ homes for 8 weeks. The PWA and CP were present for all sessions, which were designed to actively engage them both in discussion and problem solving focused on strategy use in their conversations, using video feedback alongside written materials. Session 1 introduced conversation and the impact of agrammatism, session 2 explored turns and turn construction, and session 3 repair. The next two sessions facilitated the selection (from a set of suggestions) of up to three strategies for the PWA (session 4) and the CP (session 5) to practice. Facilitation techniques included reflection on short video clips of their own conversations, to help a dyad identify positive points and challenges, and consider what they could have done differently (i.e., encourage a focus on strategy use). Session 6 explored topic, including who initiates topics and how. The final sessions (7 and 8), focused on active practice of each person’s chosen strategies in structured conversation activities. Where possible, these were videotaped to allow immediate reflection and feedback on the consequences of conversational behaviors. An overview of sessions grouped by main aim is provided in the Supplementary Material (Appendix 1). The free BCA electronic resource^1^ provides further detail about the intervention, and video-recorded examples of therapy sessions.

All therapy for all dyads was carried out by the fourth author (FB) of the study. Prior to therapy, the project team met to view baseline videos recorded by a dyad, in order to identify key barriers and facilitators to conversation. The barriers and facilitators used by each dyad are listed in the Supplementary Material (Appendix 2). These determined the choice of video clips that were prepared for therapy, which formed the basis of discussion and dyad self-reflection. While the project team guided a dyad’s reflection in this way, the final decision on which strategies to practice in therapy rested with the PWA and CP.

The intervention was tailored via the use of individual dyads’ own conversation videos, and by giving each dyad a choice concerning strategies for practice. However, therapy plans for each session detailing generic activities congruent with underlying session aims were followed throughout. Thus, once the study had begun there was no modification to the intervention schedule or principles (TIDieR checklist item 9, Hoffmann et al., 2014).

#### Fidelity of Intervention

In order to investigate how closely the actual delivery of the therapy corresponded to that which had been planned (Tate et al., 2013), aspects of TF were explored using data from seven dyads (see Heilemann et al., 2014). A rater independent of the SLP (the third author, CH) observed 23% of the 56 videotaped therapy sessions and used a BCA-specific fidelity tool in order to quantify TF. The main focus of this investigation related to two components typically associated with the concept of TF: adherence to therapy components (i.e., fidelity to the BCA session plans) and therapy delivery (i.e., therapist behavior desirable for carrying out the BCA therapy). The fidelity tool reflected these concepts in two sections: (a) procedural and (b) qualitative. In (a), the items were rated on a scale of 1, 0.5 or 0, corresponding to therapy content that was judged as being “fully”, “partly” or “not delivered” in a session, and in (b), the items were rated 1, 0.5 or 0 corresponding to statements about desired therapist behavior judged to be observable “most of the time”, “occasionally”, or “not at all”.

### Experimental Design and Processes

**Figure 1:**
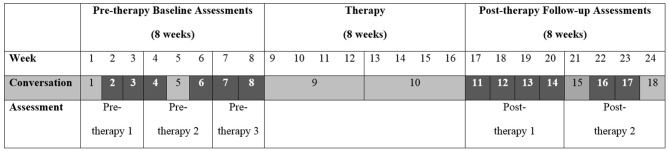
**Design of intervention study.** A total of 18 conversation samples were collected over the course of the study (eight pre-therapy, two during therapy and eight post-therapy). The conversations highlighted in the figure, six pre-therapy and six post-therapy samples, were analyzed. The figure illustrates the timing of pre-therapy baselines 1–3 and post-therapy assessments 1 and 2.

The design of the study is illustrated in Figure 1. Justification is provided under rationale for design, below, and a full explanation of the conversation sampling can be found under outcome measurement. In addition to conversations, as shown on the Figure, formal assessments were carried out both pre and post intervention: sentence comprehension (CAT; Swinburn et al., 2004), written naming (Kay et al., 1992) and digit span forward (CAT; Swinburn et al., 2004).

#### Rationale for Design

The study employed a pre-post design with multiple conversations and assessments both pre and post intervention. Use of an experimentally stronger crossover design was considered inappropriate for two reasons. First, participants were involved in the study for 6 months on average and commitments beyond this were deemed too much to ask, particularly given the requirement for the involvement throughout of CPs, several of whom were working. Second, the reliance on the goals and strategy choices of the participants themselves and the expected generalization to everyday conversation meant it was not possible to focus first on one aspect of conversation and then cross over to another aspect, as this design would require. Impairment focused intervention was not considered a suitable control as it would have been likely to have resulted in aims contrary to those of the conversation intervention in, for example, drawing attention to aspects of language such as word-finding or sentence production rather than optimizing conversation. There was no randomization to different conditions. Experimental control (Howard et al., 2015) was exerted in the following ways. First, there was ongoing contact during the pre-therapy phase which included time with the SLP, a range of language activities and questions about aphasia and conversation. This contact provided control for any Hawthorn effect (aka SLP “charm”), as change due to general SLP contact would be observed over the baseline phase rather than after therapy. Second, assessment of digit span was carried out before and after therapy. This measure provided control for any generalized effect of intervention (for a review see Murray et al., 2016). If this was occurring, change would be predicted to occur on this sensitive task, which was not at floor or ceiling for any participant. Third, all conversations were transcribed, analyzed and scored by raters blind to point of data collection avoiding any bias that could occur if pre-post therapy status of the samples was revealed.

#### Outcome Measurement

All language assessments were carried out by the fourth author (FB) of the article (Table 1 provides details).

**Table 1:** **Participant data, background information and language profiles**.

Dyad number	Age at recruitment (gender)	Months since onset of aphasia (at time of 1st session)	Previous employment	CP relation to PWA	Pyramids and Palm Trees (out of 52)*^1^	Spoken word to picture matching (out of 40)*^2^	Minimal pairs discrimination (out of 40)*^3^	Naming objects (averaged across 3 time points; out of 10)*^4^	Naming actions (averaged across 3 time points; out of 10)*^5^	Severity: Quartiles*^6^
**Dyad 1**	49 (f)	33	Jazz singer	Twin	50	38	40	8.33	2.67	3 (48%)
**Dyad 2**	39 (m)	30	Own business	Wife	52	36	40	8.33	5.67	2 (66%)
**Dyad 3**	55 (m)	59	Senior sales manager	Wife	51	39	40	9.00	2.33	3 (37%)
**Dyad 4**	63 (m)	60	Team manager NHS	Partner	48	35	29	3.33	2.00	3 (31%)
**Dyad 5**	57 (f)	39	Cashier at bookmakers	Son	51	24	30	5.33	2.00	3 (42%)
**Dyad 6**	60 (m)	17	Gardener/book illustrator	Wife	50	39	38	4.00	5.00	2 (53%)
**Dyad 7**	71 (f)	40	Deputy head teacher	Daughter	49	35	34	4.00	3.67	3 (28%)
**Dyad 8**	57 (m)	10	Self-employed van driver	Wife	44	33	31	4.33	1.33	4 (24%)

*^1^Pyramids and Palm Trees (Howard and Patterson, 1992); *^2^PALPA 47 (Kay et al., 1992); *^3^PALPA 4 (Kay et al., 1992); *^4^and *^5^Object and Action Naming Battery (OANB; Druks and Masterson, 2000); *^6^The severity measure is derived from five language subtests, carried out during the first pre-assessment and chosen to represent known areas of difficulty for people with agrammatism: OANB (Druks and Masterson, 2000), Comprehension of written sentences (CAT; Swinburn et al., 2004), Writing single words to dictation (PALPA 53, Kay et al., 1992), Spoken sentence comprehension (VAST; Bastiaanse et al., 2002). Tests and subtests which showed ceiling (e.g., Pyramids and Palm Trees) and floor effects (e.g., VAST sentence production) were excluded. The severity rating is computed in quartiles, with 4 representing most severe. Further details of performance of these tests are available from the authors. f = female, m = male. For the communication partners (CPs), gender is transparent, except dyad 1 (female) and dyad 4 (male).

The variability inherent in conversation data (Perkins et al., 1999) means it is necessary to take multiple measures to reveal patterns present over and above noise, which may be occurring for reasons including participant variables (such as fatigue) and conversation variables (such as topic). Conversation assessment entailed video recording eight pre- and eight post-therapy samples and two conversations during therapy (18 in total, henceforth labeled C1–C18). Each conversation sample lasted up to 20 min. Each dyad was trained in how to operate a digital video camera. The research SLP was not present when recordings were made. Dyads were asked to record their conversations at a time when they would normally sit down and talk, for example, to catch up on events and news. Specific topics for conversation were not proposed in an attempt to make samples as ecologically valid as possible.

Counts were made of barrier and facilitator behaviors in 5-min video samples from a subset of the 18 conversations recorded. We discarded C1, the first conversation, and C18, the final post therapy sample in order to avoid any effects of the dyad settling in to recording conversations or being aware that the study was ending. Of the remaining 14 samples, 12 were rated, six pre therapy (C2–4 and C6–8) and six post therapy (C11–14 and C16–17). C5 and C15 were only used to replace missing pre-/post-therapy samples, respectively. Where a sample was 10+ min in length, sampling began from 5 min into the recording. If a conversation was less than 10 min in length, the final 5 min were used.

The counts result from the work of 17 student SLPs (15 graduate and 2 undergraduate) at University College London between 2009 and 2013 and one PhD student (the fifth author (FJ), an experienced SLP), 18 students in total. Each year, between two and six students performed ratings as part of a thesis supervised by core members of the project team (the first (WB), second (JM) and final authors (SB)). All were trained to identify conversational behaviors in the video samples. Depending on the focus and scope of each thesis, students rated between 2 and 10 samples (with an equal number taken from pre- and post-therapy data) for between two and four dyads. For each year-group of students, training led by the final author was spread over 9 months and consisted of: a 3 h introduction to CA; a 2 h transcription training session including practice with language disordered video data; a 1 h introduction to the project; a 1.5 h introduction to the outcome measure, with discussion of written guidelines giving definitions and examples of conversational behaviors, and practice identifying turns in a short video clip of aphasic conversation; a homework task to rate the same video clip for a sample of conversation behaviors; a 2 h follow-up to give feedback on their homework ratings and to practice with a second video clip, and two support workshops (totaling 3 h). In addition, students had access to a group email address permitting them to post queries to the project team and their fellow student raters as and when they wished. During the training and rating process, the written rating guidelines were modified as necessary in response to student requests for clarification, and a revised version was emailed to all students. The rating process was as follows: student raters viewed their 5-min video samples repeatedly, and then produced a turn-by-turn orthographic transcription of each sample. They then rated the sample for core conversation behaviors using the video recording alongside the transcript, and with the support of the guidelines. Importantly at the time of rating, all students were “blind” as to all sample collection dates. Overall for each barrier and facilitator total there were 96 counts (eight dyads, 12 conversations) with between two and six barriers and 7–14 facilitators contributing to each of these counts. These variations arose as a result of the individualized choices made by each dyad.

Of the 96 conversations rated, 40 (41.7%) were analyzed by more than one student; for each of the eight dyads, at least four conversation samples were analyzed by a pair of students. In these cases, after independently rating the same sample, the student pairs agreed counts for individual behaviors. Agreement was defined as both students applying the same rating to a specific behavior; consensus agreement techniques were not used. These behaviors have no natural denominator and so percentage agreement was applied (see Hartmann, 1977). IRR was measured by averaging the IRR for each behavior and then, in turn, averaging across all barrier and all facilitator items. This measure of agreement includes counts where a behavior was agreed to occur and counts where a behavior was agreed to be absent.

#### Statistical Methods

Data from student projects were compiled by the third author (CH) under the supervision of the first (WB) and final authors (SB). Where difficulties in collating information from the student projects arose, they were resolved by discussion with the wider team (e.g., we agreed to use a mean count if there was more than one count available from different student projects for a specific behavior). The question of whether there was change with therapy was evaluated statistically for the group by taking the mean of pre-therapy scores and mean of post-therapy scores on each measure and comparing these employing a paired-sample *t*-test. This method was chosen in consultation with a statistician and in order to match the comparison made for the structured assessment tasks with that for the conversation variables. For digit span and language assessments, raw scores were entered. For conversation variables the data were first transformed by taking the square root of raw scores in order to normalize the distribution. Effect sizes were calculated using G Power version 3.1^2^.

For the case series the conversation data were analyzed using Poisson Trend Tests suitable for observations occurring in a Poisson distribution. All pre-therapy conversations were weighted the same as one another, as were all post therapy conversations. The outcomes for different participants were investigated using a Test for Homogeneity, significance demonstrated that the effects for different dyads cannot be centered on mean with variability only by chance, i.e., that there are real differences across participants. Where the Test for Homogeneity was significant the effect for different dyads was calculated by employing the Holm-Bonferroni procedure^3^. Thus the statistical procedures did not investigate trends over time but examined whether there was a difference between pre- and post-therapy scores.

## Results

### Participants

We successfully recruited nine dyads to the study (henceforth D1–D9, or PWA1–PWA9 where referring solely to the participant with aphasia). Eight dyads completed the full intervention program; the ninth withdrew half way through therapy because they felt it was not suitable for them. Table 1 provides an overview of the participant background information and scores on formal assessments. The findings are presented for the dyads in the order in which they were recruited.

PWA and their CPs varied in age and in their relationship to one another. All participants with aphasia were more than 17 months post onset at the start of their involvement in the study. On formal language assessments participants showed a range of abilities congruent with a diagnosis of agrammatic aphasia. Semantic processing of pictures as assessed by the three picture version of Pyramids and Palm Trees was near ceiling for all but PWA8. A wider range of scores was obtained on the input tasks tapping single word comprehension (word-to picture matching) and auditory discrimination (minimal pairs). Naming of objects and actions was impaired for all PWA and, for some, notably PWA1, PWA4 and PWA5, was further impaired by elements of verbal dyspraxia (based on clinical judgement; dyspraxia was not formally assessed). With regard to severity, no PWA had a score in the first or last quartile. Examples of narrative and sentence production are provided in the Supplementary Material (Appendix 3), in the form of transcriptions of the Dinner Party cartoon strip narrative (Fletcher and Birt, 1983).

### Fidelity of Treatment

To investigate IRR of the fidelity tool, 20% of the sessions rated by the first rater (the third author) were observed and rated by a second rater (a trained student SLP). There was an overall percentage agreement of 86.8% and 87.5% for the procedural and the qualitative sections of the fidelity tool, respectively.

In terms of delivery of the planned components (section (a) of the fidelity tool), results indicate a high overall adherence of the SLP to the components of BCA (fidelity score: 91.9%, SD = 3.9; based on 227 observations). For section (b) of the tool, qualitative aspects of therapy delivery, a high degree of desired therapist behavior was achieved during BCA sessions (96.7%, SD = 4.1). For a detailed description of dyad-specific and overall fidelity results see Heilemann et al. (2014).

### Inter-Rater Agreement on the Conversation Measure

The overall percentage IRR for facilitators was 69% (SD = 21) and for barriers 64% (SD = 27).

### Performance on Repeated Formal Assessments

**Figure 2:**
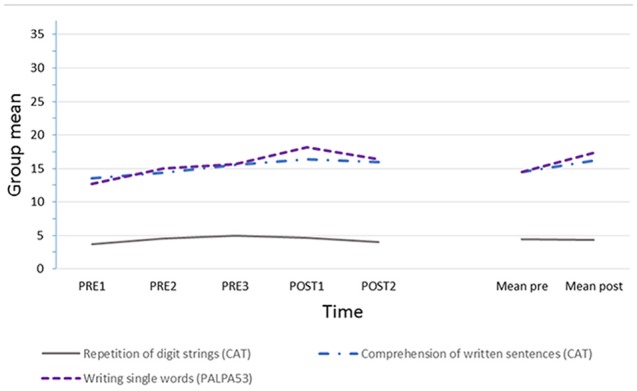
**Group means over time on: CAT comprehension of written sentences (*n* = 32); PALPA writing single words (*n* = 30); CAT repetition of digit strings (readers are referred to the manual for full scoring detail, *n* = 7 items, maximum score is 14).** Missing data: digit string repetition post therapy 2 for person with aphasia 1 (PWA1), and written naming throughout for PWA7 and PWA8 who chose not to attempt this task.

Figure 2 illustrates the mean scores for the group of PWA on the repeated language assessments and digit span test. Full details of scores for each participant at each assessment point are provided in the Supplementary Material (Appendix 4).

Visual analysis suggests relative stability in short term memory but gradual change over the course of the study for the group, with a tendency for greater change over the therapy phase in written naming. Statistical analysis, carried out on pre vs. post scores to align the comparison with that for the conversations, shows no significant change in digit span from pre to post therapy (mean pre 4.42 (SD 3.13), post 4.25 (SD 3.28), paired-sample *t*-test *t*_(7)_ = 0.247, n.s., two-tailed, effect size Cohen’s *d* = 0.05). In contrast there was a small but significant change from pre to post therapy in average performance in written naming (pre 14.44 (SD 6.02), post 17.50 (SD 4.81), paired-sample *t*-test, *t*_(5)_ = 3.051, *p* = 0.028, two-tailed, Cohen’s *d* = 0.55), and also in comprehension of written sentences (pre 14.46 (SD 4.33), post 16.63 (SD 4.27), *t*_(7)_ = 2.573, *p* = 0.037, two-tailed, Cohen’s *d* = 0.50).

### Change in Use of Conversation Facilitators

**Figure 3:**
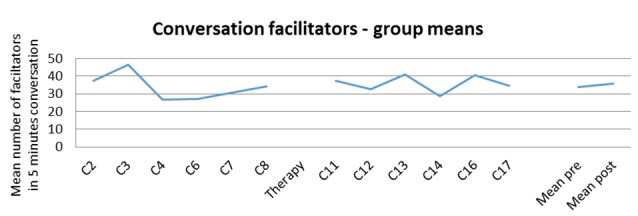
**Counts of facilitator behaviors, mean for group across the study, and pre and post therapy**.

The change in use of conversation facilitators for the group over the course of the study is illustrated in Figure 3. These facilitators include, for example, multi-modal turns (mime, gesture, drawing and writing/sky-writing) for PWA, and turns where the CP responds without the need for repair, or lets the conversation continue so the PWA can use strategies. There was no significant change in facilitators between pre and post therapy for the group (mean pre 33.72, post 35.70, paired-sample *t*-test on square-rooted values, *t* = 0.73 (7), two-tailed *p* = 0.492, n.s., Cohen’s *d* on raw values = 0.14, minimal).

The counts for conversation facilitators for each of the dyads separately are provided in Table 2.

**Table 2:** **Counts of facilitator behaviors for each dyad at each conversation**.

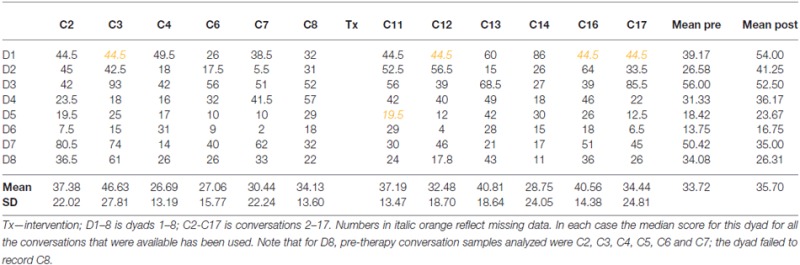

The raw figures demonstrate considerable variability in facilitator behaviors both across occasions for the same dyad, and between different dyads. The dyads also show large differences in pre-post scores. This was supported by a significant test for homogeneity (*H* = 60.52, df = 7, *p* < 0.0001) meaning the *z* scores for change calculated using the Poisson Trend Test are not centered on a mean score with variability only by chance, but that there are real differences in change between the dyads. Follow-up analysis, exploring this and employing the Holm-Bonferroni procedure, showed statistically significant change with an increase in facilitator behaviors for D2 (*z* = 4.36, *p* < 0.00,001) and D1 (*z* = 3.76, *p* < 0.001) with D5 showing a trend to significance (*z* = 1.98, *p* < 0.047), but *p* < 0.0125 is necessary for significance once the Holm-Bonferroni procedure is applied. Dyad 7 showed a statistically significant decrease in facilitator behaviors (D7 *z* = −4.09, *p* < 0.00,001) and Dyad 8 a trend in the same direction (*z* = −2.45, *p* < 0.025, but *p* < 0.01 is required when the Holm-Bonferroni procedure is applied). D3, D4 and D6 did not show significant change between pre- and post-therapy samples in either direction.

### Change in Use of Conversation Barriers

**Figure 4:**
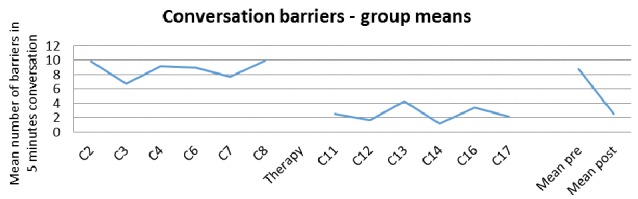
**Counts of barrier behaviors, mean for group across the study, and pre and post therapy**.

Figure 4 illustrates the marked and significant decrease in the number of barriers for the group (mean pre 8.73, post 2.52, *t* = test on square-rooted values, *t* = 2.71 (7), two-tailed *p* = 0.015, sig., Cohen’s *d* = 0.73, medium). The change reflects a reduction in number of barriers to less than a third of the number present before therapy.

The counts for conversation barriers for each of the dyads separately are provided in Table 3.

**Table 3:** **Counts of barrier behaviors for each dyad at each conversation**.

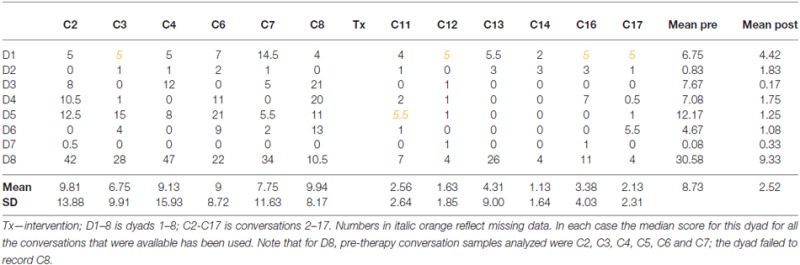

The case series data shows that, as for facilitators, considerable variability is evident in barrier behaviors and that dyads show very large differences between one another in mean pre-post scores. This was supported by a significant test for homogeneity (*H* = 95.39, df = 7, *p* < 0.0001) demonstrating the *z* scores for change calculated using the Poisson Trend Test are not centered on a mean score with variability only by chance, but that there are real differences in change between the dyads. Follow-up analysis employing the Holm-Bonferroni procedure, showed statistically significant change with a decrease in barrier behaviors for, ordered by *z* score, the following dyads: D8, D5, D3, D4 and D6 (D8 *z* = −8.24, *p* < 0.01, D5 *z* = −7.30, *p* < 0.01, D3 *z* = −6.65, *p* < 0.01, D4 *z* = −4.4, *p* < 0.025, D6 *z* = −3.66, *p* < 0.025). D1, D2 and D7 did not show significant decrease or increase in barrier use in conversation.

## Discussion

This article aimed to investigate the answers to three key questions. First, we explored whether performance on language tasks and digit span changed after conversation intervention using video feedback. The group demonstrated fairly stable performance on digit span throughout the study, with no statistically significant change between pre- and post-therapy scores. This suggests there was no change in auditory-verbal short term memory. If there had been progress, this may have reflected continued recovery or an effect of therapy and in this case the task would not have provided experimental control (Howard et al., 2015). Participants were not consistently at floor or ceiling on this task, which was selected with care, so there was scope for improvement. The stability, in spite of this, suggests the group were not within a phase of spontaneous recovery and that the intervention did not influence their short term memory/phonological processing. In this sense, it provided good experimental control, although future studies could employ multiple measures of short-term memory as advised by Murray et al. (2016).

In contrast to the stability in digit span, the group showed significantly better written naming after intervention than before, and also better comprehension of written sentences. In both cases the changes, while statistically significant, were numerically small (correct written names were provided for three additional items and sentence comprehension improved by two items, on average) and it was only for written naming that there looked to be a tendency for change to occur over the intervention period. The result might be due to the intervention as the facilitatory behaviors targeted included use of writing or “sky-writing” (i.e., tracing letters in the air). Thus, a tentative conclusion would be that conversation intervention of the type used in this study may also result in improvement in language processing on tasks that tap into processing routes employed by the strategies targeted in conversation (see Beeke et al., 2014, 2015; for a more detailed mixed methods exploration of the writing strategy for three of the dyads in this study: D6, and D4 and D8, respectively). This finding could be explored in future research by using a range of language tasks and making specific predictions as to change according to the nature of the conversation intervention for each dyad.

Second, we asked whether the number of conversation facilitators after intervention differed from that before intervention. There was no significant change in the number of facilitators for the group (minimal numerical increase from 33.7 to 35.7 on average in 5 min of conversation). This is a new finding and it stands alone as, while there are some well controlled case studies in the literature (Carragher et al., 2015), there is no research examining change at a group level in conversation behaviors after an intervention with a direct focus on conversation. The differing findings for the experimentally controlled case series are important in highlighting the variability between dyads making up the group. The significant test for homogeneity reflects the fact that there are genuine differences in change. When employing rigorous statistical analysis with an inbuilt Holm-Bonferroni procedure to adjust for multiple dyads, we found that there was a significant increase in facilitators for two of the dyads (D1 and D2). The changes for D2 are considered using a conversation analytic method in Beeke et al. (2011). D7 showed a statistically significant *decrease* in facilitator behaviors. This is important as it shows that intervention may not always operate in the desired direction. Further qualitative analysis is necessary to shed light on the nature of this statistically significant change. The study is novel in exploring the effects for the group and separately for the case series, and in demonstrating that findings for the participants may differ. Given the heterogeneity present in aphasia profiles, conversations (Perkins et al., 1999) and response to intervention, it is important for future studies of conversation-based interventions to present and analyze findings at both levels.

Finally, the study investigated whether the number of conversation barriers after intervention differed from that before intervention. For the group there was a significant and dramatic reduction from more than eight to less than three barrier behaviors on average in 5 min of conversation. This is a considerable change and one which, scaled up to the conversations between regular conversation partners throughout the day, is likely to have a large impact on everyday communication. The variability between the dyads is again reflected in the data from the case series. Here, for five dyads (D3, D4, D5, D6 and D8) there was a statistically significant reduction in barrier behaviors. The learning process that may underlie this change for PWA3 is considered in Beckley et al. (2013), and the changes for D4 and D8 are considered in detail in Beeke et al. (2015), and for D6 in Beeke et al. (2014). The remaining three dyads showed no change in barrier use in either direction. Thus, for facilitators as for barriers, the finding for the group is not the same as that for the separate dyads, although in this case there was no significant increase in barrier use (i.e., no change in the opposite direction to that targeted in intervention).

The contrast in findings for facilitators and barriers is important, and suggests a link with the effects of video feedback. It may be that participants found it easier, or more salient, to reduce or terminate barrier behaviors than to develop new or increased uses of trained facilitators. In a qualitative investigation of participants’ experience of BCA therapy and conversation change, Johnson (2015) shows that realizations about the use and impact of barrier behaviors, prompted by video feedback, resonate very powerfully with participants and are often the thing they remember most. Alternatively it may be that the therapy process used in BCA to target barriers was more effective than that for facilitators. An investigation by Johnson et al. (in press) of behavioral change in BCA demonstrates that the mechanisms reportedly supporting change to facilitators are more numerous than those reportedly supporting change to barriers. Furthermore it appears that the mechanisms of change associated with facilitators may be used in more varied combinations during therapy. However, the most effective combination of mechanisms to support change in conversation barriers and facilitators is unknown at this stage. Clearly, changing conversation behaviors through conversation therapy is a complex process that will need further investigation to ensure it is optimally effective.

It is also possible that the process of measuring change in facilitators and barriers by using frequency counts influenced the contrast in findings for facilitators and barriers. Whereas barriers by their nature are expected to decrease or disappear, facilitators *may* become more frequent, but they may instead be used more effectively while not increasing significantly in numbers. For example, a PWA may use writing as a strategy both before and after intervention; the effectiveness of this strategy within conversation may alter as a result of therapy, but not be reflected in counts of writing behavior. In addition, given that facilitators represent a range of behaviors already in use in conversation, it is unclear how much of an increase needs to happen for therapy to be considered successful. To counter this difficulty, we have attempted (in other publications, see for example Beeke et al., 2014, 2015) to deploy measures of qualitative change in the use of facilitators alongside quantitative methods, but further work in this challenging area of mixed methods is warranted.

In this study video was used with both PWA and CPs to raise awareness, promote reflection on the consequences of their conversational behaviors, and to permit them to reflect immediately on the successes and failures of strategy use in practice conversations. This promoted in-depth self-reflection and, by the very nature of video feedback, rendered the task concrete and accessible to PWA, not just their CPs. Arguably it would not have been possible to discuss abstract concepts such as turns and repair without using video-playback techniques, since speakers are generally unaware of the conversational rules that govern their interactions. In addition, speakers with aphasia are likely to experience significant difficulties with expressing views on conversation behaviors in the absence of concrete examples to scaffold such discussions. As Simmons-Mackie et al. (2014) point out, more conversation therapy directed at PWA is warranted. Video feedback would seem to be a crucial element to ensure the likely success of this form of strategy training for PWA. Indeed, as the case series findings show, two PWA significantly increased their use of facilitators in conversation post therapy. Given that generalization of learnt strategies to everyday interaction in aphasia rehabilitation is an ever-present challenge, understanding the role of video feedback in conversation therapy is a priority for systematic investigation in future research.

The inclusion of IRR is relatively new in the field and brings a level of confidence to the results. However, as the IRR results for our conversation outcome measure reveal, reliably rating conversation behaviors is not straightforward. One factor that influenced IRR was the natural variability of behaviors across conversation samples and dyads, a feature of conversation first systematically noted in relation to attempts at quantification by Perkins et al. (1999). Thus some behaviors were present for all dyads whereas some were specific to one dyad only, and some behaviors occurred throughout the conversation samples whereas others occurred only sporadically. Also, there is the issue of interpretation of IRR levels for conversation data. While there is no formally recognized acceptable level of agreement for measures of naturally occurring conversation, for the application of a measure of communicative informativeness and efficiency (the Correct Information Unit or CIU analysis of Nicholas and Brookshire, 1993) to conversation, Oelschlaeger and Thorne (1999) state that 80% and above should be considered acceptable. They remark that the 90% agreement levels obtained by other CIU studies are too high for an application to naturally occurring language phenomena. In the absence of other work in this area, the 80% agreement figure is still our best benchmark. However, the Oelschlaeger and Thorne (1999) study ultimately found that reliable CIU measures could not be obtained for everyday conversation. This is not surprising given the analytic focus on transaction (of information) to the detriment of interactional features that permit the establishment and maintenance of interpersonal relationships. This calls into question whether it is sensible to take 80% as the level of acceptable agreement when rating conversation, and points to the urgent need for further investigation of the process of interpreting IRR for such data.

This study includes several “firsts” for the field of conversation therapy for aphasia. As the largest group study in the field which deploys a measure of targeted behaviors, it presents group and case series analyses of pre- and post-therapy samples of everyday conversation, the expected site of communication behavior change following this type of intervention. While 32 dyads completed the protocol for the Wielaert et al. (2014) implementation study, low IRR of a pilot outcome measure based on SLP judgement of paired video samples made it difficult to draw conclusions regarding conversation change. As Wilkinson and Wielaert (2012) and Simmons-Mackie et al. (2014) conclude, there is a dearth of larger studies providing quantitative outcome data for conversation-based rehabilitation programs. Group results often hide the improvement that can be made after intervention; in this study a case series design reveals how individual dyads can make significant gains after conversation therapy. A strength of the study is the collection and analysis of multiple pre and post-therapy conversations; this enabled patterns to emerge over and above the noise inherent in behavioral data from everyday interaction. An analysis of TF found the delivery of therapy was in line with the intervention protocol, suggesting that BCA can be delivered as intended. Furthermore, the program appears to be acceptable to PWA and CPs of different ages, educational backgrounds, dyadic relationships and severity of aphasia. Of the nine dyads referred to the study, only one did not complete it; they reported that they would prefer an impairment based intervention, specifically targeting speech output.

Results underline the need for SLPs to tailor conversation skills training, and to consider the fact that video feedback may be crucial for generalization, although it is accepted that further evidence is needed before this can be concluded to be a vital part of such interventions. The results also show that intervention can assist communication in the absence of clear and clinically meaningful improvement on standard language tests. This is important, as a key aim of work on language in clinical practice and in some research is to improve everyday communication. The finding that everyday conversations change after video intervention work that targets conversation directly, provides clear confirmation that this is another approach that can be added to those already in use by SLPs. Finally, the study reveals the possibility of improving the everyday communication skills of some PWA many months beyond the period of spontaneous recovery. Seven of the eight dyads showed significant change at case series level and these included PWA at 59 and 60 months post stroke.

We now turn to the limitations of the study and implications for future research. From the perspective of the hierarchy of evidence, the fact that the study did not have a control group is a major concern. However, it is not clear what would be a suitable control, particularly given the heterogeneous nature of both aphasia and conversation. Furthermore, matching on conversation variables would be crucial for such a design and as shown in the data in Tables 2, 3, no two dyads show similar profiles at the level of total conversation barriers and facilitators. This is also the case for individual behaviors such as use of test questions, and gesture. Given this variability, a design in which participants act as their own control is the most likely to inform understanding. Future research might employ a cross-over design comparing work on conversation with a very different intervention (e.g., training working memory, Salis et al., 2015). As cautioned in the “Introduction” Section, work on language would not be a suitable contrasting condition for research because of the potential for confusion over goals. While this limitation holds for research, in practice, when therapy is flexible, it may well be appropriate to work directly on a language skill (e.g., using cueing to aid word retrieval) and then on transferring this ability into everyday interactions (see for example Herbert et al., 2003).

While the study is one of the largest in the field, inclusion of more dyads would strengthen the research and would enable further exploration of candidacy issues (Turner and Whitworth, 2006; Eriksson et al., 2016) and of the links between patterns in pre-therapy conversations and outcome. Future studies, with more participants, could also explore in more depth the finding that it may be easier to reduce barrier behaviors than increase facilitator behaviors. This is particularly important given the clinical implications. A tentative suggestion would be that in selecting goals for conversation intervention, SLPs should facilitate PWA and CPs to select at least one barrier behavior each, rather than a set limited to facilitator behaviors. While the inclusion of additional dyads in research would enable exploration of such issues, there is a tension present. Due to inevitable resource limitations, including more participants is likely to entail a corresponding reduction in the depth and richness of data collected and analyzed for each dyad.

The study was carried out by a University based team and, while assessment and intervention took place in participants’ homes, replication of the research in the clinical setting is important, particularly as issues of implementation in practice are likely to arise and will warrant exploration. Future research could also explore issues of dosage (bearing in mind adequate time is likely to be necessary between sessions for homework and consolidation) and intensity.

The study’s findings are applicable to people with agrammatic aphasia and their conversation partners. Use of BCA principles and particularly use of video feedback in conversation should be explored in other populations including those with fluent aphasias, and primary progressive aphasia (see Volkmer, 2013) as a starting point. The related approach of Parent Child Interaction (PCI) has been shown to be effective at changing the conversations between pre-school children with language needs and their parents (Falkus et al., 2016). Future research with school age children and teenagers with language disorders combining use of video feedback with principles of BCA and PCI is also warranted, particularly as video technology is now a routine part of many young peoples’ everyday lives.

## Conclusion

In conclusion, this article adds to the evidence base for use of conversation approaches with PWA and their CPs. The dramatic decrease in barrier behaviors after eight sessions of intervention suggests change can be expected within a clinically realistic timescale. The experimentally controlled case series (with careful correction of multiple comparisons) demonstrates that findings for the group (for example the lack of a significant increase in facilitators) are not necessarily the same as those for individual dyads. This highlights the importance of reporting findings beyond the group level. Further exploration of use of video technology in conversation therapy should be a research priority.

## Author Contributions

SB was principal investigator (PI) on the study, which was conceived and designed by SB, JM and WB and based in part on SB’s doctoral research. This core team with FB created the BCA intervention and with SIE determined criteria for inclusion and assessments to be used. WB led on the quantification of aspects of conversation, the writing of this article and carried out the analysis of conversation facilitators and barriers for the group and case series. JM led the collection and compilation of connected speech data and the IRR analysis. CH led on fidelity and under the supervision of SB and WB assembled all the data from the student projects and compiled Appendices 2 and 3. CH and JM analyzed the data from the formal assessment tasks, and CH contributed substantially to the IRR analysis. FB recruited all participants to the study, assessed them and carried out the intervention. FJ analyzed conversation data from two of the dyads, contributed to the BCA online resource and carried out the qualitative analysis discussed here in relation to this study’s quantitative findings. SIE led on inclusion criteria and facilitated recruitment to the study. DH supplied the Poisson Trend Test and provided advice on statistical analysis. SB was PI for the follow-on study which funded development of the BCA online resource for rehabilitation.

## Funding

This study was funded by the UK Stroke Association (grant number TSA2007/05). Development of BCA as an online resource was funded by the UK Economic and Social Research Council (RES-189-25-0292).

## Conflict of Interest Statement

The authors declare that the research was conducted in the absence of any commercial or financial relationships that could be construed as a potential conflict of interest.

## Footnotes

^1^https://extendstore.ucl.ac.uk/product?catalog=UCLXBCA

^2^http://www.gpower.hhu.de/

^3^The Holm-Bonferroni procedure works as follows: with all eight dyads, consider the *p*-values (arising from the Poisson Trend Test) from smallest to largest. The smallest is compared with 0.05/8 and if less than this it is significant. If it significant then assess the next at 0.05/(*n* − 1), if that is significant assess the next at 0.05/(*n* − 2). The process was repeated until the result for the next dyad was not significant.
