# Supplementary material for: Conversation Therapy with People with Aphasia and Conversation Partners using Video Feedback: A Group and Case Series Investigation of Changes in Interaction
Source: Front Hum Neurosci. 2016 Nov 7;10:562. doi: 10.3389/fnhum.2016.00562 (PMC5097900; doi:10.3389/fnhum.2016.00562)
Supplement: Supplementary file 1 [file DataSheet_1.docx]

Supplementary Material

**Conversation therapy with people with aphasia and conversation partners using video feedback: a group and case series investigation of changes in interaction.**

Wendy Best^*^, Jane Maxim, Claudia Heilemann, Firle Beckley, Fiona Johnson, Susan Edwards, David Howard, Suzanne Beeke

*** Correspondence:** Prof Wendy Best: w.best@ucl.ac.uk

# Supplementary Figures and Tables

## Supplementary Figures

**Appendix 1**: Better Conversations with Aphasia therapy program: An overview of sessions grouped by main aims

## Supplementary Tables

**Appendix 2**: List of conversation barriers and facilitators for PWA and CPs (unless otherwise stated, each item was rated for all dyads). Items where the dyad appears in parentheses indicate that the behavior was a targeted therapy goal only relevant to that dyad.

|  | **Item analysis** | |
| --- | --- | --- |
|  | **Faciliators** | **Barriers** |
| **PWA-focused items** | PWA produces **two or more turns** in succession without speaker change occurring | PWA initiates **correct production sequence** (Dyad 1 only) |
|  | PWA produces **key word(s)** during turn | PWA produces **‘yes’ when means ’no’**, or vice versa (Dyad 1 only) |
|  | PWA produces **key word(s)** during **turn that contains mime** (Dyad 3 only) | PWA’s turn contains (long) **pauses** with no outward sign of intention to continue (Dyad 2 only) |
|  | PWA produces **mime** during turn | PWA **repeats the same turn** when asked for clarification by CP (Dyad 7 only) |
|  | PWA produces **gesture** during turn |  |
|  | PWA produces **drawing** during turn |  |
|  | PWA produces **(sky)writing** during turn |  |
|  | PWA **indicates a new topic** by raising index finger and saying ‘oh’ (or similar) (Dyad 7 only) |  |
| **N** | **8** | **4** |
| **CP-focused items** | CP responds **without need for repair** (exclude CP passing turns) | CP takes the floor to ask **clarification question before PWA’s turn is complete** (Dyad 1 only) |
|  | CP responds with a **passing turn** | CP asks a **test question** (one to which CP knows the answer) |
|  | CP lets the conversation **continue** (i.e. pauses for further clues/so PWA can use strategies) | CP prompts PWA to re-do her turn with **grammatical structure** (Dyad 5 only) |
|  | CP **carries on if has understood** PWA’s turn (it does not need to be perfect) | CP initiates **correct production sequence** |
|  | CP responds to PWA turn by **commenting** | CP comments on PWA’s **competence** (e.g. ‘that’s right’, ‘well done’) |
|  | CP asks **‘are you still thinking’** (or similar) during PWA mid-turn pause (Dyad 2 only) | CP’s turn is a **question** (Dyad 8 only) |
|  | CP **accepts** the meaning of PWA’s **gesture**: word not requested (Dyad 3 only) |  |
|  | CP **paraphrases** the meaning of PWA’s prior turn |  |
|  | CP **writes/draws during her (CP) turn** to aid PWA’s understanding (Dyad 7 only) |  |
| **N** | **9** | **6** |
| **Total** | **17** | **10** |
| **% PWA** | **47%** | **40%** |
| **% CP** | **53%** | **60%** |

**Appendix 3**: Samples of connected speech collected pre-intervention using the Dinner Party cartoon strip narrative (Fletcher and Birt, 1983).

| **PWA1** | **transcribed sample length 06:54 minutes** |
| --- | --- |
| PWA | uhm (pause) dinner barty (pause) uhm (pause) one (pause) hi: phone uhm lovely uhm (pause) men, yeah, dinner, phone, yep lovely thanks. |
| RA | [mmhmm] yeah? |
| PWA | yeah. uhm (pause) two, uhm (pause) uhm (pause) yeah. wife uhm, man, yeah uhm /ch/ ki-kitch-en uhm /salspins/ uhm uhm (pause) ((tuts)) (pause) fish, yeah, yeah, yes, uhm, yeah |
| RA | yep, yes you can. |
| PWA | yeah, uhm (pause) ok, uhm three, uhm (pause) /wavsh/, uhm uhm, dinner lovely, uhm, uhm, four, uhm (pause) sex, no ((laughs)), god, no uhm ((laughs)), no! uhm ((laughs)) (pause) |
| RA | take your time, it’s fine. |
| PWA | ((laughs)), oh, oh, uhm, four, hum ((laughs)) no |
| RA | compose yourself, straight face, straight face ((laughs)) |
| PWA | four, (pause) uhm (pause) uhm (pause) dinner, no uhm (pause) /lgl/ /l:/ leg-liss yeah, uhm /s/ pressure /ch/ bret ((sighs)) (pause) tie yeah man yeah (pause) yeah ((sighs)) ok? yeah? uhm drive, uhm wife yeah, /lauwers/ yeah, uhm meech mer meecher me-tilwer /w/ me ter (pause) ok |
| RA | alright, just do as much as you can |
| PWA | ok, six, yeah, uhm, ((sighs)) oh! yeah, fish, (pause) no! ((mimes)), gat yeah, yes, no! o! ((mimes)) man uhm (pause) wife wi-f- uhm, cat! (pause) ok, fish, finished, sev- uhm sev- uhm seven, uhm, wep wep. yeah? ((imitates crying)) uhm /sh/ uhm /s/ uhm /s:/ chip chip uhm fish /s/ yeah (pause) eight, uhm, thank you (pause) uhm cat, yeah, ((mimes eating, licking lips)) yeah ((laughs)) uhm, (pause) fish, j-chips, yeah. |

| **PWA2** | **transcribed sample length 02:20 minutes** |
| --- | --- |
| PWA | (pause) ready! ((laughs)) |
| RA | fantastic! yeah |
| PWA | (pause) eight o’clock (pause) Friday (pause) s:me er smiths ((laughs)) |
| RA | yeah |
| PWA | (pause) uhm (pause) uhm (pause) d-dinner uhm (pause) ba- uhm (pause) tidying up uhm ready (pause) uhm short and tie |
| RA | mmmm |
| PWA | (pause) uhm eight o’clock uhm (pause) come on in |
| RA | mhhm |
| PWA | a salmon ((laughs)) (pause) uhm running |
| RA | mmhmm |
| PWA | fish and chips ((laughs)) go back in (pause) uhm (pause) fish and chips ((laughs)) but the (pause) the cat |
| RA | mmhmm |
| PWA | uhm gone ((Laughs)) yes |

| **PWA3** | **transcribed sample length 02:57 minutes** |
| --- | --- |
| PWA | oh erm (pause) phone |
| RA | mhhmm |
| PWA | a:n, (pause) oh dinner alright? Fine yes right erm a:n erm (pause) erm cooking a:n (pause) erm washing up |
| RA | mmhmm |
| PWA | A:n (pause) ((tut)) erm (pause) erm (pause) salmon |
| RA | mmhmm |
| PWA | A:n uhm (pause) w washing up and (pause) an:n erm er erm licing a candle and (pause) uhm (pause) his erm oh erm lighting a candle a:nd (pause) uhm ((tut)) uhm one two three four |
| RA | Mmhmm |
| PWA | Uhmm (pause) and (pause) uhm uhm neccklace (pause) uhm (pause) prep through ground. Oh necklace oh yeah yeah |
| RA | Yep |
| PWA | Yeah (pause) An erm (pause) yep yep tie and shoes (pause) and (pause) erm (pause) a erm (pause) shook hands |
| RA | Yeah |
| PWA | and a flower (pause) er Mrs and Mr |
| RA | Yeah |
| PWA | and and Missa and Mrs (pause) alright? And (pause) erm (pause) ‘A gone! Oh! Oh!’ erm ‘salmon gone oh’ ((Laughs)) Oh deary deary me uhm (pause) an oh ‘I don’t know oh::, I don’t know oh:::’ Yeah anyway erm oh gone (pause) yeah er gone yeah and (pause) Fish an Chips |
| RA | Yeah |
| PWA | Erm (pause) and (pause) Uhm fish and chips yum yum yum yum yeah yeah an er cat ‘hmm yum yum yum’ ((Laughs)) |

| **PWA4** | **transcribed sample length 03:52 minutes** |
| --- | --- |
| PWA | Erm (pause) and the phone and the phone and the phone (xxxx) (pause) what |
| RA | Yeah so you can keep going yeah just ignore just keep going |
| PWA | Yeah ((Laughs)) erm (pause) he (pause) erm (pause) erm (pause) cooking erm erm (pause) plaice erm (xxxx) erm (pause) m: (pause) no ((Laughs)) erm (pause) erm I and (xxxx) huge er |
| RA | mhmm |
| PWA | Er t- the garden: erm (pause) er bored |
| RA | mhhm |
| PWA | li- li- (pause) erm (pause) er shed no no no no (pause) erm (pause) tied (pause) shoe shoes (pause) tied erm (pause) ball (pause) no (pause) er (pause) erm (pause) erm (pause) er (xxxx) flowers erm or drop erm (pause) hmm (pause) harn (gasps) hmm yeah (pause) and a cat |
| RA | ((Laughs)) |
| PWA | Erm (xxxx) erm (pause) he (xxxx) and erm (xxxx) and a dog and (xxxx) yeah |
| RA | Great |

| **PWA5** | **transcribed sample length: 01:52 minutes** |
| --- | --- |
| PWA | erm (pause) erm erm (pause) phone-uh d-one dunno erm washing yeah yeah tomorrow yeah |
| RA | yeah |
| PWA | yeah yeah oh uhm uhm oh dunno uhm (pause) uhm uhm (pause) err uhm no dunno dunno err uhm (pause) uhm yeah yeah uhm no uhm yeah err (pause) oh dunno no |
| RA | take your time alright |
| PWA | no |
| RA | not a rush |
| PWA | rush uhm (pause) flowers yeah uhm dunno dunno erm (pause) dunno yeah dunno |
| RA | have a look |
| PWA | fish and chips |
| RA | mmhmm |
| PWA | yeah fish and chips uhm cat |
| RA | mmhmm |
| PWA | and uhm oh uhm uhm erm (pause) uhm no dunno no |

| **PWA6** | **transcribed sample length 08:36 minutes** |
| --- | --- |
| PWA | rone the (pause) is erm (pause) paper paper no (tut) er no it’s a (tut) (pause) uh um (pause) going to go t-to town |
| RA | mhhm right |
| PWA | the (pause) the (pause) g-going to go (pause) fis fish wish we’re going to going (hhh) |
| RA | yeah |
| PWA | the table go in (pause) i-in (pause) the cat going to the their erm table cough cough (pause) erm the (pause) well the (pause) is erm (tut) (pause) erm err oh (pause) is going to go (pause) no it’s |
| RA | that’s alright |
| PWA | well, welton welton in th- in the going (pause) well in (pause) is (pause) is in erm (xxxx) (pause) welton in garden in the garden |
| RA | yeah |
| PWA | in the going (pause) in (pause) is erm well in (pause) /k/ kevin catherine k- no (pause) (tut) (hhh) paper paper no (tut) (pause) (hhh) ((points at fish in pic 2)) |
| RA | yeah |
| PWA | well is (pause) /s/ (hhh) (pause) (hhh) we erm (xxxx) water water no (pause) (hhh) walker wal- erm (hhh) (pause) (hhh) in the in the |
| RA | yeah |
| PWA | in the in the (pause) going going to the w-wis: (hhh) going to going (pause) chits: (tut) (hhh) going to (hhhh) (pause) in (pause) in the going to going (pause) well (pause) (hhh) well in oh er (pause) (hhh) no |

| **PWA7** | **transcribed sample length 05:25 minutes** |
| --- | --- |
| PWA | erm (pause) writing |
| RA | yeah |
| PWA | erm (pause) cooking gir-girl boy ok uh boy boy |
| RA | mmhmm yeah |
| PWA | uhm fishing err drying erm (pause) washing washing uhm (pause) c-cooking (pause |
| RA | great |
| PWA | uhm (pause) boy uhm (pause) clicking (pause) uhm eggs (pause) uhm (pause) wine uhm napki-napkin wine |
| RA | mmhmm |
| PWA | uhm (pause) cooking (pause) table. girl (pause) sicking-sitting uhm (pause) dog (pause) |
| RA | mmhmm |
| PWA | flowers (pause) girl boy (pause) boy girl shanking hands uhm (pause) coat! |
| RA | great |
| PWA | ok (pause) oh cat! cat uhm (pause) wine (pause) survelwear (pause) uhm napkin |
| RA | mmhmm |
| PWA | horror-horror! oh my god! horror! (pause) crying |
| RA | yeah |
| PWA | oh dear (pause) uhm girl boy (pause) uh-uhm (pause) ((sighs)) |
| RA | you’re doing a good job here |
| PWA | ok uhm fish and chip uhm s- (pause) chips (pause) boy boy running (pause) |
| RA | great |
| PWA | ((points at cat and laughs)) err cat |
| RA | mmm |
| PWA | licking ((laughs)) uhm (pause) girl boy (pause) girl (pause) uhm |

| **PWA8** | **transcribed sample length: 02:11 minutes** |
| --- | --- |
| PWA | dinner |
| RA | mmhmm |
| PWA | ((whistles)) (pause) dinner uhm oh yeah (pause) |
| RA | take it one bit at a time so just look at- you were on this one weren’t you? |
| PWA | mmm |
| RA | tell me what’s happening in that picture |
| PWA | ((draws circle)) plate |
| RA | yeah |
| PWA | p-late |
| RA | yeah |
| PWA | uhm (pause) ((sighs)) (pause) yeah (pause) fing fingers ((laughs)) look look look fish fingers fucking hell ((sings: “I don’t know what’s going on today”)) uhm dinner yeah (xxxx) |
| RA | yeah |
| PWA | alright err uhm chish and chips |
| RA | yeah |
| PWA | right (pause) erm (pause) oh yeah (pause) yeah look unbelievable (xxxx) ((laughs)) look look look (pause) yeah one two right |
| RA | yeah |
| PWA | (pause) fish fingers absolutely |

**Appendix 4**: Repeated tasks, full data - digit span, written naming, sentence to picture matching.

|  | | **Repetition of digit strings (CAT)**  **(out of 14)** | **Comprehension of written sentences (CAT)**  **(out of 32)** | **Writing single words (PALPA53)**  **(out of 30)** |
| --- | --- | --- | --- | --- |
| **Pre 1** | PWA1 | 6 | 18 | 13 |
|  | PWA2 | 4 | 14 | 23 |
|  | PWA3 | 6 | 19 | 1 |
|  | PWA4 | 0 | 9 | 10 |
|  | PWA5 | 0 | 12 | 16 |
|  | PWA6 | 0 | 18 | 13 |
|  | PWA7 | 10 | 7 | N/A |
|  | PWA8 | 4 | 11 | N/A |
|  | **Mean (SD)** | **3.75 (3.62)** | **13.50 (4.5)** | **12.67 (7.23)** |
| **Pre 2** | PWA1 | 4 | 16 | 17 |
|  | PWA2 | 0 | 20 | 22 |
|  | PWA3 | 4 | 20 | 5 |
|  | PWA4 | 0 | 13 | 14 |
|  | PWA5 | 6 | 9 | 17 |
|  | PWA6 | 4 | 20 | 15 |
|  | PWA7 | 12 | 7 | N/A |
|  | PWA8 | 6 | 10 | N/A |
|  | **Mean (SD)** | **4.50 (3.82)** | **14.38 (5.37)** | **15.00 (5.62)** |
| **Pre 3** | PWA1 | 6 | 18 | 16 |
|  | PWA2 | 4 | 20 | 23 |
|  | PWA3 | 4 | 20 | 7 |
|  | PWA4 | 4 | 13 | 15 |
|  | PWA5 | 0 | 11 | 19 |
|  | PWA6 | 6 | 18 | 14 |
|  | PWA7 | 12 | 14 | N/A |
|  | PWA8 | 4 | 10 | N/A |
|  | **Mean (SD)** | **5.00 (3.38)** | **15.50 (4.00)** | **15.67 (5.35)** |
| **Post 1** | PWA1 | 3 | 23 | 19 |
|  | PWA2 | 6 | 20 | 24 |
|  | PWA3 | 4 | 21 | 8 |
|  | PWA4 | 6 | 14 | 20 |
|  | PWA5 | 0 | 13 | 21 |
|  | PWA6 | 4 | 17 | 17 |
|  | PWA7 | 10 | 11 | N/A |
|  | PWA8 | 4 | 12 | N/A |
|  | **Mean (SD)** | **4.63 (2.88)** | **16.38 (4.53)** | **18.17 (5.49)** |
| **Post 2** | PWA1 | N/A | N/A | N/A |
|  | PWA2 | 6 | 22 | 20 |
|  | PWA3 | 4 | 19 | 10 |
|  | PWA4 | 0 | 12 | 18 |
|  | PWA5 | 0 | 10 | 21 |
|  | PWA6 | 0 | 17 | 13 |
|  | PWA7 | 12 | 16 | N/A |
|  | PWA8 | 6 | 16 | N/A |
|  | **Mean (SD)** | **4.00 (4.47)** | **16.00 (4.04)** | **16.40 (4.72)** |
